# *Solving Not Answering*. Validation of Guidance for Writing Higher-Order Multiple-Choice Questions in Medical Science Education

**DOI:** 10.1007/s40670-024-02140-7

**Published:** 2024-08-20

**Authors:** Maria Xiromeriti, Philip M. Newton

**Affiliations:** Swansea University Medical School, Swansea University, Singleton Park Campus, Swansea, Wales, SA2 8PP UK

**Keywords:** Digital education, Bloom’s taxonomy, Assessment, Higher-order learning

## Abstract

Problem-solving and higher-order learning are goals of higher education. It has been repeatedly suggested that multiple-choice questions (MCQs) can be used to test higher-order learning, although objective empirical evidence is lacking and MCQs are often criticised for assessing only lower-order, factual, or ‘rote’ learning. These challenges are compounded by a lack of agreement on what constitutes higher order learning: it is normally defined subjectively using heavily criticised frameworks such as such as Bloom’s taxonomy. There is also a lack of agreement on how to write MCQs which assess higher order learning. Here we tested guidance for the creation of MCQs to assess higher-order learning, by evaluating the performance of students who were subject matter novices, vs experts. We found that questions written using the guidance were much harder to answer when students had no prior subject knowledge, whereas lower-order questions could be answered by simply searching online. These findings suggest that questions written using the guidance do indeed test higher-order learning, and such MCQs may be a valid alternative to other written assessment formats designed to test higher-order learning, such as essays, where reliability and cheating are a major concern.

**Supplementary Information:**

The online version contains supplementary material available at 10.1007/s40670-024-02140-7.

## Introduction

The use of multiple-choice questions (MCQs) to assess higher order learning is standard practice for assessing clinical knowledge in the health professions [1, 2]. However, the creation of such questions is not straightforward, often requiring ‘Item Writing Workshops’ to train staff in the creation of clinical scenarios for such questions [3, 4]. There is a need to expand the use of higher order MCQs, beyond the evaluation of clinical scenarios and into medical science education more generally, where assessments of ‘higher order learning’ may be currently given in the form of written coursework such as essays and dissertations. These assessments tend to show limited coverage of the curriculum and are vulnerable to a number of forms of misconduct, such as plagiarism [5], outsourcing to commercial writers [6], and the use of Chatbot AI tools such as ChatGPT [7].

Many authors have written guidance for the use of MCQs to assess higher order learning, and these were recently reviewed into a set of guidance principles for educators wishing to write their own questions [8]. A key feature of MCQs that assess higher-order learning is the use of problem-type scenarios, which the learner then solves, rather than the recall of a standalone fact [9]. The scenario should contain a lot of information, as is the case in real-world problems, and the test-taker has to critically appraise the information to identify key relevant features [10]. Another feature of MCQs which assess higher order learning is the use of ‘assumed knowledge’; i.e., the student is required to have specific subject knowledge or skills which are missing from the problem scenario and thus can act as a cognitive bridge between the problem/question and the answer options [11]. Students who do not have this knowledge will not be able to answer the question.

Higher order learning is usually defined with reference to Bloom’s taxonomy or some other hierarchy (e.g. [1, 2, 9, 11–14]). Bloom’s taxonomy [15, 16] is usually presented as a hierarchy of verbs for the creation of learning outcomes, with the principle that outcomes created using verbs from the base of the hierarchy (e.g. list, recall) are ‘lower order’ learning, whereas verbs at the higher end of the taxonomy (e.g. ‘evaluate’, ‘justify’) represent ‘higher order learning’ [17]. However, Bloom’s taxonomy has faced many decades of criticism from multiple angles, including a concern that it cannot meaningfully identify ‘higher order learning’ [18–21], and recent studies in the UK and the USA have shown that the presentation of Bloom’s taxonomy is remarkably inconsistent between universities and other sources. That is, one presentation of Bloom’s may place a verb at the very bottom of the taxonomy, whereas another will place that same verb at the very top [17, 22].

Despite these criticisms, it is still common for Bloom’s taxonomy to be used as the reference point when defining higher order assessment items. One approach is to ask participants (e.g. academics or students) to assign assessment items to the relevant level of Bloom’s taxonomy, or to identify relevant action verbs when writing the items [1, 11, 23]. These sorts of validations were undertaken in the studies which were reviewed to develop the higher-order item writing guidance being tested here [8]. However, this sort of validation normally requires collapsing the six tiers of the taxonomy into just two or three, and then asking subject experts to make subjective ratings about the level of learning which is being assessed by the question. Even then it has been repeatedly shown that academics find it difficult to do this reliably (e.g. the ratings of an assessment item as ‘higher’ or ‘lower order’ will vary between academics) [24–27]. Other taxonomies of learning have also been used to classify learning by cognitive level; for example, the ‘Structure of Observed Learning Outcomes’ (SOLO) Taxonomy, popular in higher education in the UK, has been used to design MCQs which aim at assessing ‘higher order’ learning [28], while in North America, clinical question banks may be classified into 1st, 2nd, and 3rd orders, with 1st-order questions assessing factual recall, while 3rd-order questions require critical thinking and problem-solving [29, 30]. Again though, while there are guidelines for the creation of questions which map to these levels, there is a paucity of objective evidence showing that the assigning of questions to these levels is reliable.

An alternate approach is to generate objective data about the ability of students to correctly answer the questions, dependent on their level of expertise, on the basis that students with existing lower order knowledge will find it easier to answer the higher-order questions than subject-novices who do not have that knowledge. These hypotheses are supported by some prior research (e.g. [23]), but still the majority of research in this area appears to rely on subjective ratings of question difficulty. Here then, we tested guidance designed to help educators write higher-order MCQs [8], by generating objective data on the ability of subject novices vs experts to answer questions which had been written using the guidance.

## Methodology

### Guidance for the Creation of Higher Order MCQs

This guidance has been published previously, in a detailed form [8], based on a number of papers describing the use of MCQs to assess higher order learning [10, 12, 14, 31–46]. A summary of the guidelines is shown in Table 1. Examples of higher order questions written using the guidance, and their lower order equivalents, are given in ESM Appendix 1.

**Table 1:** Summary of principles for the creation of higher-order bridge MCQs. Derived from [8]

• ***Start with a lower order MCQ that assesses factual knowledge.*** Identify an existing question, or write a simple MCQ that tests factual knowledge
• ***Problem scenario***. (Re)write the question stem into a problem that needs to be solved rather than a question to be answered. One simple way to do this is to write a scenario that describes the correct answer, but in non-technical terms
• ***Identify assumed knowledge (bridge).*** The student should have some prior knowledge in order to answer the question, without which the question cannot be answered. This prior knowledge must be relevant to interpreting both the question and the answer options. One way to identify assumed knowledge is to identify a sequence of steps/knowledge needed to solve a problem, and take out those in the middle [33]
• ***Use common language***. Using everyday language, as a more authentic problem presentation. Avoid technical terms, as these can signpost correct answers. Where technical terms are unavoidable, try to put them in only one of either the scenario, or the answer options
• ***Use active answers***. Having the answer options as actions, or describing actions, makes them less likely to be a list of facts
• ***Use annotated images.*** (optional)
• ***Number of answers***. Increase these to ~ 8, to allow for more granularity in the answer options

We conducted two different sets of experiments to compare questions written using the guidance with their lower order counterparts. A summary of the key differences between these experiments is given in Table 2.

**Table 2:** Summary of the two sets of experiments conducted here

Feature	Experiment 1	Experiment 2
*Aim*	Pilot proof of principle	Full testing of guidance
*Participants*	Novices only	Novices vs experts
*Lower order questions*	Author generated	Existing questions from textbooks
*Payment*	Flat fee	Flat fee plus bonus per question
*Answer options*	Different between higher order	Consistent between higher order and lower order
*Subject*	Neuroscience	Genetics

All studies were carried out using Qualtrics surveys with student participants recruited via the online labour market Prolific (www.Prolific.com). The fee was at an estimated hourly rate of £8, advised by Prolific to be a ‘good’ rate at the time of the study. Participants were screened into expert or novice groups on the basis of the subjects being studied at university. The full list of subjects which were used to screen participants for each study is shown in ESM Appendix 1. Before beginning the study, all participants were given information about their data protection rights, their right to withdraw at any time, and a contact email for any questions. We were not aware of any similar studies in the literature which might give us a reasonable expectation of the possible sizes of any differences between the experimental groups, and so we were unable to undertake a meaningful power analysis and sample size calculation. Thus, the sample sizes are based on the experience of the authors.

*Ethical Approval.*

Both experiments were approved by the ethics committee of the Swansea University Medical School ref SUMS RESC 2022-0042A.

### Experiment 1. Novices Only, Proof of Principle

An initial study was conducted to determine whether subject matter novices could meaningfully answer lower order questions, under time-limited conditions. In this same experiment, we then evaluated whether this ability would be reduced when the questions were rewritten using the guidance from Table 1. Questions were tested on novice students, meaning they were studying subjects unrelated to the subject matter of the questions (neuroscience). Each question was asked in both higher and lower-order formats, and under both closed book and open book conditions.

### Procedure

We evaluated the performance of the questions under two conditions: ‘closed book’ wherein participants were asked to try and answer the questions without referral to any other sources, and then an ‘open book’ condition, wherein students were encouraged to ‘cheat’ by any means, for example by using Google or Alexa. (These experiments were conducted in Summer 2022, prior to the launch of ChatGPT.) Participants were asked to ‘cheat’ rather than to use ‘an open book condition’ on the basis that this would be more familiar terminology and so easier for the participants to correctly follow the instructions. To minimise confusion in the instructions for participants, each participant was asked to answer in only one condition (closed or open book), but was asked questions from both formats (lower order or higher order), although the format difference was not explained. Thus, a participant only saw one version of an individual question. The same questions were asked, in the same order, in each group, but in different formats (higher order or lower order). Therefore, the experimental groups were as follows:

1. Higher-lower (closed book)
2. Lower-higher (closed book)
3. Higher-lower (open book)
4. Lower-higher (open book)

Thus, groups 1 and 3 would start with Q1 in a higher-order format, followed by Q2 in a lower-order format, then Q3 in a higher order format, and so on. Groups 2 and 4 would start with Q1 in a lower-order format, Q2 in a higher order format, and so on. Dropout and motivation were a concern, since it seemed likely that participants would be unable to answer the questions, and so the study was conducted in two parts: an initial proof-of-principle pilot evaluated the performance of 12 questions, and then the second part evaluated the performance of 5 further questions. Twelve participants were recruited to each group, except for group 3 which had 13 participants in the initial pilot, due to a technical error. The tasks on Prolific were set so that any one participant could only be recruited to one study.

**Fig. 1:**
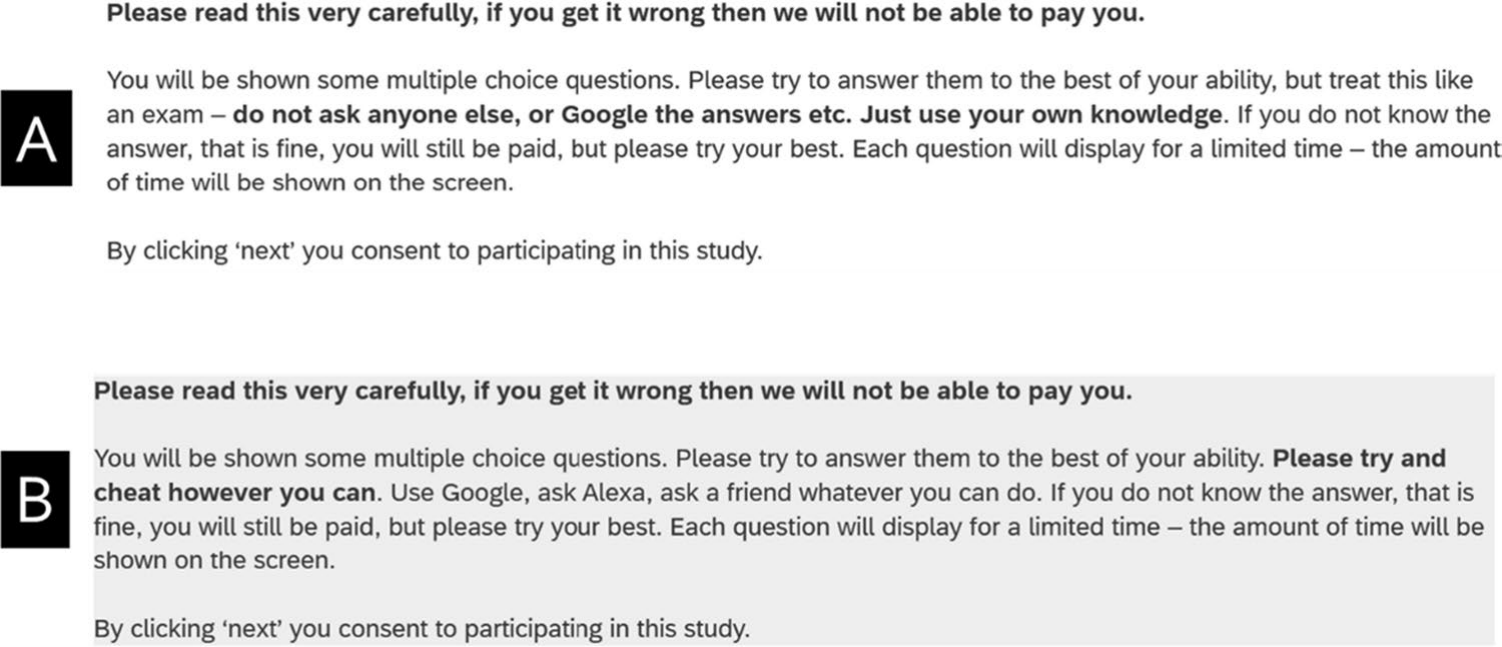
Instructions given to participants in **A** the closed book condition and **B** the open book condition experiment of Experiment 1

The task was advertised on Prolific as ‘A Study about University Assessment Methods’. Participants were given the instructions shown in Fig. 1.

The participants were then given a maximum of 90 s to answer each question, after which the instrument automatically forwarded on to the next question. At the end of the survey, the purpose of the study was explained in more detail and those who had participated in the open-book group were asked what methods they had used.

### Experiment 2. Novice vs Expert, Testing of Full Guidance

The first experiment used both lower and higher order questions written by the authors, which potentially leads to a conflict when we also designed the research. Experiment 2 was designed to test the guidance in a more general context that reflects the practical application of the guidance, by starting with lower-order questions that had *not* been written by the authors, but which subject novices could obtain the correct answer through simple Googling. These questions were identified as described below, and then rewritten by the authors following the guidance in Table 1. We hypothesised that, once rewritten subject experts would still be able to answer the questions, but novices would not, even in the open book condition. In Experiment 1, participants were also not provided with any specific motivation to find the correct answer in the open book condition. We did not want participants to simply give up, especially since this seemed more likely for novices and so could artificially bias the data. This was addressed in Experiment 2 by giving participants a small financial incentive for answering correctly under the open book condition. A final difference between the two studies was that, in Experiment 1, the answer options were different between some of the lower and higher order formats of the same question, in accordance with the guidance (e.g. to make the answer options active). Here we retained the same answer options with the lower and higher order versions, including keeping the same correct answer. This was to ensure that the revised questions were assessing the same learning outcome as the original lower-order question, although it did present a challenge with utilising some aspects of the guidance (e.g., to make the answer options active, and to present them in plain language).

A pilot study was undertaken to identify ‘lower order’ questions which novices could only answer under open-book conditions. Twenty questions were selected from the introductory chapter of a genetics textbook [47] and from past-papers of the United Kingdom Biology A-level exam (these are the exams taken by students aged ~ 18, in part as the basis for entry to higher education). They were selected by the authors on the basis that they should be questions whose answers would be reasonably familiar to current undergraduates studying the life sciences.

**Fig. 2:**
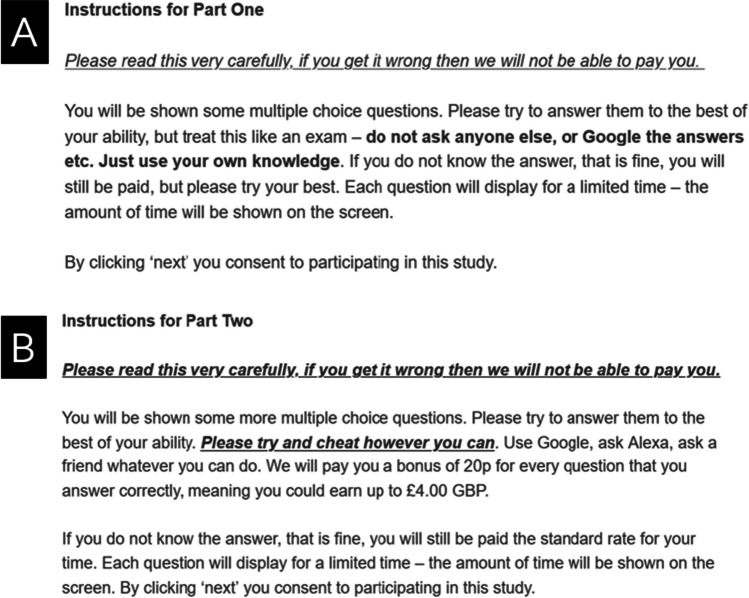
Instructions given to all participants at **A** the beginning (closed book) and **B** halfway (the open book condition) of Experiment 2. For the second part of Experiment 2, only 10 questions were used, and so the bonus payments and instructions identified in section **B** were modified accordingly

Participants were then given the instructions shown in Fig. 2.

Twenty participants were recruited to each of four groups. This experiment also included two attention-check questions, one in each condition. These were questions which appeared to be regular multiple-choice questions formatted in the same way as others in the study, but where the question stem included an instruction to select a specific answer. In keeping with the Prolific.co guidance on attention checks, participants who failed both attention checks by selecting the incorrect answer were not paid and their data were not included. Additional participants were then added as required.

1. Novices, closed book
2. Novices, open book
3. Experts, closed book
4. Experts, open book

Using these pilot data, we identified questions for further development, based upon the following criteria:

- Large difference in performance on open book vs closed book for novices (i.e. questions in which the open book condition allowed the participants to score highly)
- Experts scored highly under both conditions
- Experts scored higher than non-subject specialists under the closed book condition, to ensure the expertise tested was not common knowledge.

The precise metrics for identifying questions were not formalised—each question was considered individually by both authors, and selected questions were then drafted into the higher order format using the guidance in Table 1. Both authors drafted higher-order questions and then a final version of each question was agreed through discussion. The experiment was then repeated but with ten higher-order questions.

### Analysis

For both experiments, analysis was undertaken by question, with the dependent variable being the percentage of participants who answered a question correctly. Specific statistical tests are identified in the relevant section of the results.

For the methods used by students in the open book condition, a simple quantitative content analysis [48] was performed on the very brief free-text comments left by participants.

Example questions from Experiment 1 and Experiment 2 are shown in ESM Appendix 1 (the full set of questions is available upon reasonable request, from the corresponding author). Statistical analysis and figure creation were undertaken using GraphPad Prism V10 (San Diego, CA).

## Results

### Experiment 1

**Fig. 3:**
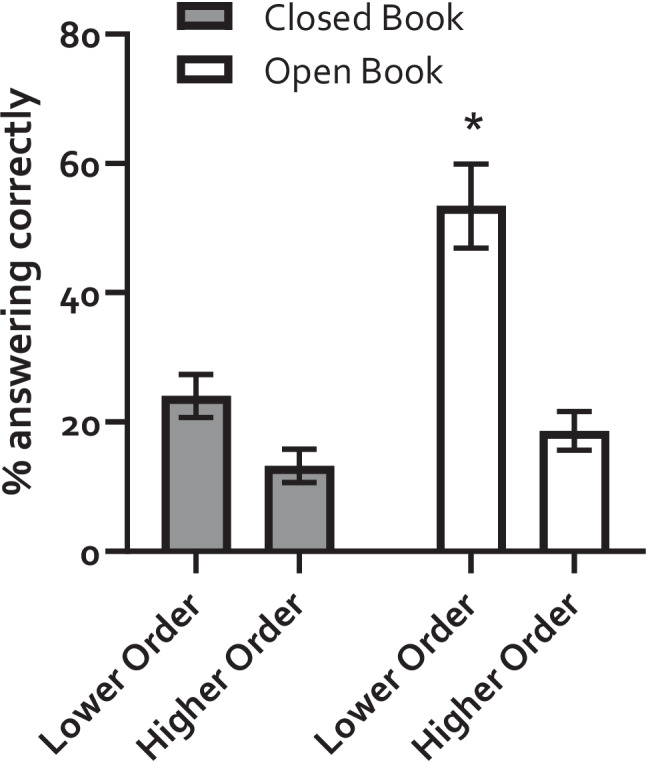
Rewriting lower order questions into higher order makes it harder for subject matter novices to answer. Different groups of participants were given the same question in two different formats, lower order and higher order, and under two different conditions, open book and closed book. Participants were able to answer the lower order questions in the open-book condition but were not able to answer the questions in the closed-book condition or when they were rewritten into the higher order format, even under the open-book condition. *P, 0.05 when compared to all other conditions by post hoc Bonferroni tests following two-way repeated measures ANOVA (see text for details)

There was a clear effect of condition, where an average of 53.4% of participants were able to answer the questions in the open-book condition when the questions were written in the lower order format, compared to 18.6% when the questions where in the higher order format. In the closed book condition, an average of 24% of participants answered correctly in the lower order format with 13.2% answering correctly in the higher format. The percentage of participants able to answer each question in the lower order, open-book format was significantly higher than for every other format/condition when analysed using a two-way repeated measures ANOVA, with percentage of participants answering correctly as the dependent variable, and test format (closed book vs open book) and question format (higher order vs lower order) as the conditions. There was a significant effect of question format (*F* (1, 16) = 25.03, *P* = 0.0002), and test format (*F* (1, 16) = 14.59, *P* < 0.0001) and a significant interaction between the two (*F* (1, 16) = 6.95, *P* = 0.0018). Post hoc Bonferroni tests revealed a significant difference between the lower-order, open-book condition, and all other conditions. No other significant differences were observed. The results are shown in Fig. 3.

### Methods Used in the Open-Book Condition

In 25 participants, 22 left comments, most very brief (the total corpus was 542 words). All 22 identified ‘searching the internet’ as their strategy, with 20/22 naming Google directly, and one asking ‘Siri’. In 22 participants, 6 identified the time limit as a factor which made it difficult to answer the questions. In 22 participants, 4 identified that there were questions that were more ‘difficult’ (the participants were not told that the questions were in two different formats).

## Experiment 2

The results from Experiment 1 were clear: novices used Google to successfully answer questions written in a lower-order format, but this approach was not successful when the questions were in a higher-order format. However, the design of the study contained some potential confounds; (1) the participants had no obvious motivation to try and answer correctly. This was potentially more significant in the higher order condition due to the extra work required to successfully answer the question, and the increased length and complexity of the questions. (2) Both lower and higher-order questions were written by the authors. (3) There is no within-study positive control since Experiment 1 only used subject matter novices, and thus it is not clear that subject experts could still answer the higher-order questions. This is important to demonstrate that the rewritten questions remain a valid form of assessment for the subject matter content. Finally, the answer options were often completely different between the lower order and higher order question formats.

Thus, in Experiment 2, we included a within-experiment positive control. We also started with existing lower order questions, in the public domain, that had *not* been written by either of the authors, using the original answer options and same correct answer, but with additional answer options added.

**Fig. 4:**
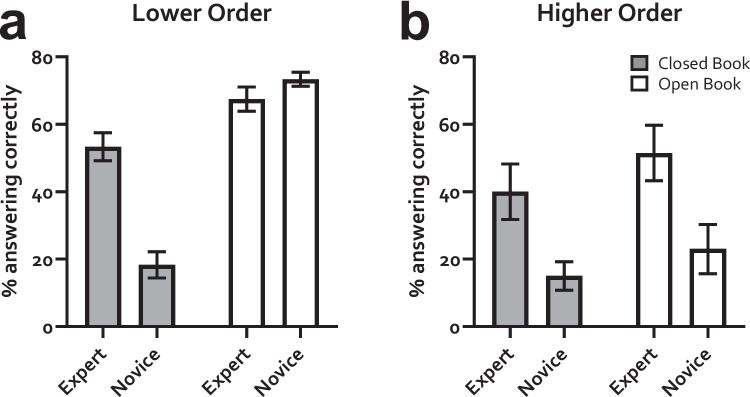
Rewriting publicly available lower order questions into higher order questions makes it harder for novices to answer them, but experts can still answer under closed-book conditions. Different groups of participants were given the same question in two different formats, lower order and higher order, and under two different conditions, open book and closed book. Novices were able to answer the lower order questions in the open-book condition but not when the questions when rewritten into a higher order format using the guidance tested here. Experts showed good ability to answer questions under all conditions. See text for statistical analysis

The results are shown in Fig. 4. Novices were again able to successfully answer lower order questions by Googling the answers, an average of 73.3% answering correctly but this dropped to 23% when the questions were rewritten into a higher order format. A two-way repeated measures ANOVA was conducted, with percentage of participants answering correctly as the dependent variable, and test format (closed book vs open book) and expertise (novice vs expert) as the conditions. For lower order questions (Fig. 3A), there was a significant effect of question format (*F* (1, 9) = 163.0, *P* < 0.0001) and expertise (*F* (1, 9) = 17.09, *P* = 0.0025), with a significant interaction between these two conditions (*F* (1, 9) = 61.91, *P* < 0.0001). A post hoc Bonferroni multiple comparisons test showed a significant effect of expertise in the closed-book condition (*P* < 0.0001) but not the open-book condition (*P* = 0.2931). For the higher order questions (Fig. 3B), a two-way repeated measures ANOVA again showed a significant effect of question format (*F* (1, 9) = 6.553, *P* = 0.0307) and expertise (*F* (1, 9) = 18.40, *P* = 0.0020), but no interaction between the two conditions (*F* (1, 9) = 0.2687, *P* = 0.6167).

If the higher order questions are truly assessing higher order learning, then they should be harder, and so the percentage of experts answering them would be lower. An average of 53.3% of experts were able to answer questions in the lower order format under closed book conditions; this dropped to 40% when rewritten into the higher order format. This same comparison was 67.5% and 51.5% in the open book format. When analysed using a paired *t-*test, the difference between lower and higher order was significant for both the closed book (*t* = 3.234, df = 16, *P* = 0.0052) and the open book condition (*t* = 4.740, df = 16, *P* = 0.0002).

## Discussion

Here we conducted a validation test on guidance which is designed to help educators write higher order MCQs [8]. When questions were written using a traditional lower order format, subject-matter novices were able to Google their way to the correct answer, to the same extent as experts, even though the novices were studying subjects unrelated to the topic. When those same questions were rewritten into a higher-order format using the guidance, subject-novices found it significantly harder to answer using Google, while experts were still able to answer the questions. These findings suggest that questions written using the summary guidance in Table 1 do indeed assess higher-order learning. The summary guidance in Table 1 is largely complementary to, and intended to be used alongside, a large body of existing literature on what makes an effective multiple-choice question, regardless of whether they assess lower or higher-order learning [39, 49, 50].

There are a number of factors and potential limitations that need to be considered when interpreting our findings.

The guidance shown in Table 1 contains a number of different elements which combine to make the question an active, problem-solving exercise. On the basis of this study, it is not currently possible to determine which, if any, of the individual elements is the most important for ensuring that an MCQ assesses ‘higher order learning’. This is the subject of ongoing work where each element is subject to a systematic experimental appraisal. These analyses may result in a revised and condensed set of guidelines which could prioritise individual elements for the creations of higher-order MCQs.

When using online labour markets for opinion surveys, there is potentially an issue participants could simply give random, or minimal, answers [51], or that the participants may in fact be ‘bots’ [52]. Here we had objective outcome measures with right and wrong answers. Although there was no incentive to answer correctly, we did see a clear and expected difference in those experimental conditions which would indicate that participants are real, valid, and following instructions (for example between subject experts and novices).

Participants in the open-book conditions were instructed to ‘cheat’ by using whatever sources necessary. Even so, subject novices struggled to answer the higher order questions. However, it is important to be clear that this is not specifically a study of ‘cheating’ and that these higher-order questions are not ‘cheat-proof’, especially in the new era of ChatGPT, which can answer very complex problem-solving MCQs [53], and when cheating in online open-book exams is already high [54, 55]. Indeed, the ability of ChatGPT to answer questions written using these guidelines has been recently tested and ChatGPT answers almost all the questions correctly, apart from where novel, labelled images are included [56]. However, these higher order questions should be more resilient to cheating in invigilated examinations, and offer a way to assess higher order learning in such exams. In this way, a supervised exam based on these questions would likely be more resistant to misconduct than other assessment formats which are currently used to assess higher order learning, such as essays and other asynchronous written coursework; these are open to multiple sources of misconduct such as plagiarism [5] contract cheating [6] and can be completed to a high standard using tools such as ChatGPT [7].

Guidelines developed here have been tested in only two subject areas, both of which are from human physiology and medical science. It seems reasonable to propose the guidelines could be used to test higher order learning in other subjects, including those outside of medical science education. This will require careful validation and is also the subject of future work.

In summary, we have validated guidance for item-writing, or rewriting, based on the literature for the use of MCQs to assess higher order learning. In an experimental situation, questions that were rewritten using this guidance were much more challenging to answer by simple Googling but were still answerable by students who were studying relevant subjects, although those experts found the questions harder. These findings suggest that the guidance could be used by educators and institutions to develop MCQ-based exams to assess higher order learning, to partially replace asynchronous written coursework such as essays.

## Supplementary Information

Below is the link to the electronic supplementary material.

Supplementary file1 (DOCX 18 KB)

## References

[1] Kim M-K, Patel RA, Uchizono JA, Beck L. Incorporation of Bloom’s taxonomy into multiple-choice examination questions for a pharmacotherapeutics course. Am J Pharm Educ [Internet]. 2012 [cited 2022 Apr 8];76. Available from: https://www.ajpe.org/content/76/6/114 10.5688/ajpe766114 PMC3425929 22919090

[2] Bibler Zaidi NL, Grob KL, Yang J, Santen SA, Monrad SU, Miller JM, et al. Theory, process, and validation evidence for a staff-driven medical education exam quality improvement process. Med Sci Educ. 2016;26:331–6.

[3] Dellinges MA, Curtis DA. Will a short training session improve multiple-choice item-writing quality by dental school faculty? A pilot study J Dent Educ. 2017;81:948–55. 28765439 10.21815/JDE.017.047

[4] Parkes J. Maybe brief multiple-choice question workshops are good enough. J Fac Dev. 2021;35:20–6.

[5] Foltýnek T, Meuschke N, Gipp B. 2019 Academic plagiarism detection: a systematic literature review. ACM Comput Surv. 52:112:1-112:42

[6] Newton PM. How common is commercial contract cheating in higher education and is it increasing? A systematic review. Front Educ [Internet]. 2018 [cited 2022 Jun 27];3. Available from: https://www.frontiersin.org/article/10.3389/feduc.2018.00067

[7] Herbold S, Hautli-Janisz A, Heuer U, Kikteva Z, Trautsch A. AI, write an essay for me: a large-scale comparison of human-written versus ChatGPT-generated essays [Internet]. arXiv; [cited 2023 May 8]. Available from: http://arxiv.org/abs/2304.14276 10.1038/s41598-023-45644-9 PMC10616290 37903836

[8] Newton PM. 2023 Guidelines for creating online MCQ-based exams to evaluate higher order learning and reduce academic misconduct. In: Eaton SE, editor. Handb Acad Integr [Internet]. Singapore: Springer Nature; [cited 2023 Jul 13]. p. 1–17. Available from: 10.1007/978-981-287-079-7_93-1

[9] Billings M, DeRuchie K, Hussie K, Kulesher A, Merrell J, Morales A, et al. Constructing written test questions for the health sciences [Internet]. National Board of Medical Examiners; 2020. Available from: https://www.nbme.org/sites/default/files/2020-11/NBME_Item%20Writing%20Guide_2020.pdf

[10] Joorabchi B. How to…:construct problem-solving MCQs. Med Teach. 1981;3:9–13. 24476031 10.3109/01421598109081736

[11] Palmer EJ, Devitt PG. Assessment of higher order cognitive skills in undergraduate education: modified essay or multiple choice questions? Research paper BMC Med Educ. 2007;7:49. 10.1186/1472-6920-7-49 PMC2148038 18045500

[12] Kar SS, Lakshminarayanan S, Mahalakshmy T. Basic principles of constructing multiple choice questions. Indian J Community Fam Med. 2015;1:65.

[13] Kolomitro K, MacKenzie LW, Lockridge M, Clohosey D. Problem-solving strategies used in anatomical multiple-choice questions. Health Sci Rep. 2020;3:e209. 33305012 10.1002/hsr2.209 PMC7714269

[14] MacFarlane L-A, Boulet G. Multiple-choice tests can support deep learning! Proc Atl Univ Teach Showc. 2017;21:61–6.

[15] Bloom BS, Krathwohl DR. Taxonomy of educational objectives. The classification of educational goals, Handbook I: Cognitive domain. New York: Longmans Green; 1956.

[16] Krathwohl DR. A revision of Bloom’s taxonomy: an overview. Theory Pract. 2002;41:212–8.

[17] Newton PM, Da Silva A, Peters LG. A pragmatic master list of action verbs for Bloom’s taxonomy. Front Educ [Internet]. 2020 [cited 2020 Jul 14];5. Available from: https://www.frontiersin.org/articles/10.3389/feduc.2020.00107/full

[18] Ormell CP. Bloom’s taxonomy and the objectives of education. Educ Res. 1974;17:3–18.

[19] Pring R. Bloom’s taxonomy: a philosophical critique (2). Camb J Educ. 1971;1:83–91.

[20] Sockett H. Bloom’s taxonomy: a philosophical critique (I). Camb J Educ. 1971;1:16–25.

[21] Stedman CH. An analysis of the assumptions underlying the taxonomy of educational objectives: cognitive domain. J Res Sci Teach. 1973;10:235–41.

[22] Stanny CJ. Reevaluating Bloom’s taxonomy: what measurable verbs can and cannot say about student learning. Educ Sci. 2016;6:37.

[23] Mate KE, Weidenhofer J. Are online examinations a viable alternative to paper-based examinations for assessment of human physiology? Proc Aust Conf Sci Math Educ. 2021;78–83.

[24] Dempster ER, Kirby NF. Inter-rater agreement in assigning cognitive demand to life sciences examination questions. Perspect Educ. 2018;36:94–110.

[25] Karpen SC, Welch AC, Cross LB, LeBlanc BN. A multidisciplinary assessment of faculty accuracy and reliability with Bloom’s taxonomy. Res Pract Assess. 2017;12:96–105.

[26] Karpen SC, Welch AC. Assessing the inter-rater reliability and accuracy of pharmacy faculty’s Bloom’s taxonomy classifications. Curr Pharm Teach Learn. 2016;8:885–8.

[27] Pollitt A, Ayesha A, Crisp V. The demands of examination syllabuses and question papers. Tech Monit Comp Exam Stand. 2007;166–206.

[28] Davies GR, Proops H, Carolan CM. The development and use of a multiple-choice question (MCQ) assessment to foster deeper learning: an exploratory web-based qualitative investigation. J Teach Learn. 2020;14:1–12.

[29] Deebel NA, Terlecki R. ChatGPT performance on the American Urological Association Self-assessment Study Program and the potential influence of artificial intelligence in urologic training. Urology. 2023;177:29–33. 37209880 10.1016/j.urology.2023.05.010

[30] Copeland-Halperin LR, O’Brien L, Copeland M. Reply to comment: Evaluation of artificial intelligence-generated responses to common plastic surgery questions. Plast Reconstr Surg Glob Open. 2023;11(11):e5454. 38025641 10.1097/GOX.0000000000005454 PMC10662903

[31] Aiken LR. Writing multiple-choice items to measure higher-order educational objectives. Educ Psychol Meas. 1982;42:803–6.

[32] Azer SA. Assessment in a problem-based learning course: twelve tips for constructing multiple choice questions that test students’ cognitive skills. Biochem Mol Biol Educ. 2003;31:428–34.

[33] Burns ER. “Anatomizing” reversed: use of examination questions that foster use of higher order learning skills by students. Anat Sci Educ. 2010;3:330–4. 21046570 10.1002/ase.187

[34] Di Giusto F, Müller Werder C, Reichmuth A, Adams-Hausheer D, Christian J. Multiple-choice questions: teaching guide for higher and professional education. 2019 [cited 2022 Jun 28]; Available from: https://digitalcollection.zhaw.ch/handle/11475/19339

[35] DiBattista D. Making the most of multiple-choice questions: getting beyond remembering. Collect Essays Learn Teach. 2008;1:119–22.

[36] Domyancich JM. The development of multiple-choice items consistent with the AP Chemistry curriculum framework to more accurately assess deeper understanding. J Chem Educ. 2014;91:1347–51.

[37] Douglas-Morris J, Ritchie H, Willis C, Reed D. Identification-based multiple-choice assessments in anatomy can be as reliable and challenging as their free-response equivalents. Anat Sci Educ. 2021;14:287–95. 33683830 10.1002/ase.2068

[38] Fuhrman M. Developing good multiple-choice tests and test questions. J Geosci Educ. 1996;44:379–84.

[39] Haladyna TM. Writing test items to evaluate higher order thinking. Allyn and Bacon; 1997.

[40] Maryani I, Prasetyo ZK, Wilujeng I, Purwanti S, Fitrianawati M. HOTs multiple choice and essay questions: a validated instrument to measure higher-order thinking skills of prospective teachers: research article. J Turk Sci Educ. 2021;18:674–90.

[41] Prevost LB, Lemons PP. Step by step: biology undergraduates’ problem-solving procedures during multiple-choice assessment. CBE-Life Sci Educ. 2016;15(4):71. 10.1187/cbe.15-12-0255 PMC5132368 27909021

[42] Pugh D, De Champlain A, Gierl M, Lai H, Touchie C. Can automated item generation be used to develop high quality MCQs that assess application of knowledge? Res Pract Technol Enhanc Learn. 2020;15:12.

[43] Qureshi SY. How does washback of different formats of assessment work within classroom in physical sciences- a holistic study. Sci Educ Int [Internet]. 2018 [cited 2022 Oct 21];29. Available from: http://www.icaseonline.net/journal/index.php/sei/article/view/108

[44] Scully D. Constructing multiple-choice items to measure higher-order thinking. Pract Assess Res Eval [Internet]. 2017 [cited 2022 Jun 28];22. Available from: https://eric.ed.gov/?id=EJ1143362

[45] Wilkie RM, Harley C, Morrison C. High level multiple choice questions in advanced psychology modules. Psychol Learn Teach. 2009;8:30–6.

[46] Young A, Shawl SJ. Multiple choice testing for introductory astronomy: design theory using Bloom’s taxonomy. Astron Educ Rev. 2013;12.

[47] Solomon B. Medical genetics and genomics [Internet]. Wiley Blackwell; 2022 [cited 2022 Dec 20]. Available from: https://blackwells.co.uk/bookshop/product/Medical-Genetics-and-Genomics-by-Benjamin-D-Solomon/9781119847182

[48] Stemler S. An introduction to content analysis. ERIC Digest [Internet]. ERIC Clearinghouse on Assessment and Evaluation, 1129 Shriver Laboratory, University of Maryland, College Park, MD 20742; 2001 Jun. Available from: https://eric.ed.gov/?id=ED458218

[49] Haladyna TM, Downing SM. A taxonomy of multiple-choice item-writing rules. Appl Meas Educ. 1989;2:37–50.

[50] Haladyna TM, Rodriguez MC. Developing and validating test items [Internet]. 1st ed. Routledge/Taylor & Francis Group; 2013 [cited 2022 May 6]. Available from: https://www.routledge.com/Developing-and-Validating-Test-Items/Haladyna-Rodriguez/p/book/9780415876056

[51] Anduiza E, Galais C. 2017 Answering without reading: IMCs and strong satisficing in online surveys. Int J Public Opin Res. 29:497–519

[52] Chmielewski M, Kucker SC. An MTurk crisis? Shifts in data quality and the impact on study results. Soc Psychol Personal Sci. 2020;11:464–73.

[53] Newton PM, Xiromeriti M. ChatGPT performance on MCQ exams in higher education. A pragmatic scoping review [Internet]. EdArXiv; 2023 [cited 2023 Jun 23]. Available from: https://edarxiv.org/sytu3/

[54] Newton PM, Essex K. How common is cheating in online exams and did it increase during the COVID-19 pandemic? A systematic review. J Acad Ethics [Internet]. 2023 [cited 2023 Aug 7]; Available from.10.1007/s10805-023-09485-5

[55] Newton PM. The validity of unproctored online exams is undermined by cheating. Proc Natl Acad Sci. 2023;120:e2312978120. 37788313 10.1073/pnas.2312978120 PMC10576111

[56] Newton PM, Summers CJ, Zaheer U, Xiromeriti M, Stokes JR, Bhangu JS, et al. Can ChatGPT-4o really pass medical science exams? A pragmatic analysis using novel questions [Internet]. medRxiv; 2024 [cited 2024 Jul 9]. p. 2024.06.29.24309595. Available from: https://www.medrxiv.org/content/10.1101/2024.06.29.24309595v2 10.1007/s40670-025-02293-z PMC12058600 40352979

