# Supplementary material for: Solving Not Answering. Validation of Guidance for Writing Higher-Order Multiple-Choice Questions in Medical Science Education
Source: Med Sci Educ. 2024 Aug 20;34(6):1469–77. doi: 10.1007/s40670-024-02140-7 (PMC11698704; doi:10.1007/s40670-024-02140-7)
Supplement: Supplementary file 1 — Supplementary file1 (DOCX 18 KB) [file 40670_2024_2140_MOESM1_ESM.docx]

**Discipline inclusion/exclusion criteria for Experiment 1**

Students studying the following subjects were eligible to participate in the study; Accounting, Agriculture and Horticulture, Archaeology, Architecture, Art and/or design, Building Planning, Business, Classics, Communication and/or Media, Computer Science, Computing (IT), Counselling, Earth Sciences, Economics, Education, Engineering, English Language, English Literature, Fashion and textiles, Finance, Geography, History, Hospitality/Catering, Languages, Law, Leisure, Management, Marketing, Materials Science, Mathematics, Music, Philosophy, Physics, Politics, Social Work, Sociology, Sports, Theatre/Dramatic Arts, Theology/Religion, Travel and Tourism.

Students studying the following subjects were excluded; Biochemistry (Molecular and Cellular), Biological Sciences, Biology, Biomedical Sciences, Chemistry, Dentistry, Genetics, Health and Medicine, Medicine, Nursing, Pharmacology, Psychology, Science, Veterinary Medicine. Students who had selected ‘Other’ as their subject were also excluded.

**Discipline inclusion/exclusion criteria for Experiment 2**

Novices were defined on the basis of studying the following subjects; Accounting, Agriculture and Horticulture. Archaeology. Architecture. Art and/or Design, Building Planning, Business, Classics, Communication and/or Media, Computer Science, Computing (IT), Counselling, Earth Sciences, Economics, Education, Engineering, English Language, English Literature, Fashion and textiles, Finance, Geography, History, Hospitality/Catering, Languages, Law, Leisure, Management, Marketing, Material Science, Mathematics, Music, Philosophy, Physics, Politics, Psychology, Social Work, Sociology, Sports, Theatre/Dramatic arts, Theology/Religion, Travel and Tourism.

Experts were defined on the basis of studying the following subjects; biochemistry, biology, biological sciences, biomedical science, genetics, medicine, science. Students in any undergraduate year of study were eligible to participate, on the basis that these were basic genetics questions that many students would have covered in their college level study prior to beginning university.

| ***Type*** | ***Lower Order*** | ***Higher Order*** |
| --- | --- | --- |
| ***Problem*** |  | *Viv feels a stinging sensation on the back of their knee. When Viv looks down, a mosquito has bitten them on the back of the knee, and now it starts to itch.* |
| ***Stem*** | *Where in the brain is the Primary Somatosensory Cortex located****?*** | *Which term best describes the anatomical location of the region of the brain that allows Viv to tell that the stinging sensation is coming from her knee?* |
| ***Bridge*** |  | 1. ***Location of Central Sulcus*** 2. ***Meaning of Anatomical Terms*** |
| ***Answer Options*** | 1. Anterior Cingulate Cortex 2. Anterior Frontal Cortex 3. Anterior Parietal Cortex 4. Anterior Temporal Cortex 5. Posterior Cingulate Cortex 6. Posterior Frontal Cortex 7. Posterior Parietal Cortex 8. Posterior Temporal Cortex | 1. Anterior to the Central Sulcus 2. Lateral to the Central Sulcus 3. Medial to the Central Sulcus 4. Posterior to the Central Sulcus 5. Anterior to the Lateral Sulcus 6. Inferior to the Lateral Sulcus 7. Rostral to the Lateral Sulcus 8. Posterior to the Lateral Sulcus |

**An example question from Experiment 1.** The Lower Order question has been rewritten using the guidance. In addition to the features described, there is common language and ‘chaff’ used in the problem and the answer options are active, in the sense that they describe a location of something relative to something else, rather than simply a series of nouns.

| ***Type*** | ***Lower Order*** | ***Higher Order*** |
| --- | --- | --- |
| ***Problem*** |  | *Whitty and Vallance are flatmates who have recently returned from a festival. They both start to feel ill, with a runny nose, fatigue and a cough. Whitty also has a fever, and a Lateral Flow Test shows that Whitty is COVID-positive. Vallance does not test positive for COVID, but instead appears to have a common cold. They both decide to isolate though, since their third flatmate, Tony, is recovering from a bout of Monkeypox.* |
| ***Stem*** | *All viruses consist of* | *The infectious agents for all three illnesses are comprised of;* |
| ***Bridge*** |  | *That COVID, Common colds and Monkeypox are all causes by a virus* |
| ***Answer Options*** | 1. DNA and a protein coat 2. RNA and a protein coat 3. A nucleic acid and a protein coat 4. Polysaccharides and proteins | 1. DNA and a protein coat 2. RNA and a protein coat 3. A nucleic acid and a protein coat 4. Polysaccharides and proteins 5. Polysaccharides and a lipid envelope 6. Nucleic acids only 7. Polysaccharides only 8. Lipids only |

**An example question from Experiment 2.** The Lower Order question, this time taken from a textbook, has been rewritten using the guidance. In addition to the features described, there is common language used in the problem.
